# Relation between Fractal Inhomogeneity and In/Nb-Arrangement in Pb(In_1/2_Nb_1/2_)O_3_

**DOI:** 10.1038/s41598-017-17349-3

**Published:** 2017-12-13

**Authors:** Shinya Tsukada, Kenji Ohwada, Hidehiro Ohwa, Shigeo Mori, Seiji Kojima, Naohiko Yasuda, Hikaru Terauchi, Yukikuni Akishige

**Affiliations:** 1 Faculty of Education, Shimane University, Matsue, Shimane 690-8504 Japan; 2 National Institutes for Quantum and Radiological Science and Technology (in SPring-8), Sayo-cho, Sayo-gun, Hyogo, 679-5148 Japan; 3 Electrical and Electric Engineering Department, School of Engineering, Gifu University, Gifu, 501-1193 Japan; 4 Department of Materials Science, Osaka Prefecture University, Sakai, Osaka 599-8531 Japan; 5 Pure and Applied Sciences, University of Tsukuba, Tsukuba, Ibaraki 305-8573 Japan; 6 Advanced Research Center of Science, School of Science, Kwansei Gakuin University, Sanda, Hyogo 669-1337 Japan; 7 Office of the Vice President for Research, Shimane University, Matsue, 690-8504 Japan

## Abstract

Relaxor ferroelectrics show substantial responses to electric fields. The key difference from normal ferroelectrics is a temperature-dependent inhomogeneous structure and its dynamics. The lead-based complex perovskite Pb(In_1/2_Nb_1/2_)O_3_ is an intriguing system in which the inhomogeneous structure can be controlled by thermal treatment. Herein, we report investigations of the phase transitions in single crystals of Pb(In_1/2_Nb_1/2_)O_3_ via changing the degree of randomness in which In and Nb occupy the B site of the ABO_3_ perovskite structure. We studied the dynamic properties of the structure using inelastic light scattering and the static properties using diffuse X-ray scattering. These properties depend on the degree of randomness with which the B site is occupied. When the distribution of occupied In/Nb sites is regular, the antiferroelectric phase is stabilised by a change in the collective transverse-acoustic wave, which suppresses long-range ferroelectric order and the growth of the inhomogeneous structure. However, when the B site is occupied randomly, a fractal structure grows as the temperature decreases below *T* ^*^~475 K, and nanosized ferroelectric domains are produced by the percolation of self-similar and static polar nanoregions.

## Introduction

Because a crystalline solid comprises a periodic arrangement of atoms, crystals are usually expected to be spatially homogeneous. However, in real materials, crystal imperfections often result in inhomogeneities such as nanodomains and nanoclusters. Such inhomogeneous systems have attracted significant attention in the field of condensed matter physics because fluctuations in the inhomogeneous structures can potentially lead to large responses to external fields^1–3^.

Relaxors are a special class of inhomogeneous systems in which mesoscopic polar regions induce giant dielectric and electromechanical responses^3–5^. Ever since the discovery of record-setting electromechanical responses in lead-oxide perovskite—Pb(B′B′′)O_3_—relaxors, these compounds have been used for applications in high-power military sonar, ultrasonic transducers for diagnosis devices and high-precision actuators^4^. The origin of the huge dielectric and electromechanical response is understood conceptually as being due to the reversible motion of mesoscopic polar domains and domain boundaries on length scales of micrometres^3–5^.

From structural considerations, the unstable domains are attributed to the arrangements of local polar regions several nanometres wide called static polar nanoregions (PNRs), which are similar to the ferroelectric nanodomains (FNDs) in a typical ferroelectric such as BaTiO_3_^6^. Therefore we can understand relaxors in the same conceptual framework as BaTiO_3_, using the Comes–Guinnier–Lambert model in which the dynamic PNRs in relaxors correspond to nanometre-sized regions with time-dependent polarization^7–10^. However, compared with BaTiO_3_, relaxors are still not clearly understood because of their complexity, which originates from fluctuating polar structures that spread over large length and time scales^11–14^. The generation of the wide distributions of characteristic lengths and timescales in relaxors can be attributed to the random electric fields produced by different ions occupying equivalent lattice sites^15,16^; the investigation of the effects of random electric fields on the distributions of the characteristic length and time scales is thus necessary.

A method for characterising the spatial structures and dynamics of relaxors may be offered by the introduction of ‘fractons’, which involves the concept of a fractal and its local vibrations. Although fractals are usually found in noncrystalline glasses, polymers and gels^17–20^, PNRs form clusters, nanodomains and microdomains, which generate fractal structures in crystalline relaxors over nanometre-sized regions^13,14,21,22^. In addition, although the fractal-and-fracton picture describes systems on a coarse-grained level, it deals well with a broad distribution of length and time scales. Thus, the fractal picture provides a powerful technique for understanding relaxors. Fractals in relaxors are reported to change near phase transitions^13,14^, but few reports exist that treat the relaxor structure and dynamics in a unified approach, even though they are connected with each other. The present study addresses this issue using quasi-elastic light scattering (QELS) and diffuse synchrotron X-ray scattering experiments on Pb(In_1/2_Nb_1/2_)O_3_ (PIN) to investigate the distribution of spatial structures and dynamics. Based on the temperature dependence of these properties, we discuss phase transitions in relaxors from the viewpoint of fractals.

**Figure 1:**
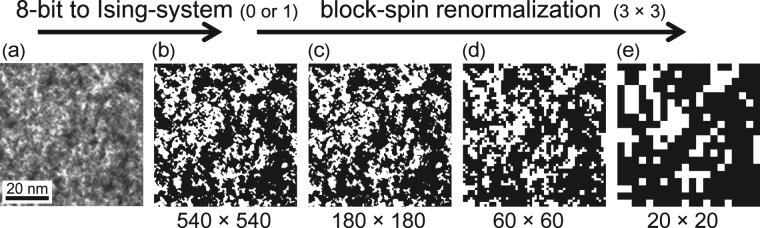
(**a**) Microstructure in D-PIN obtained from electron-diffraction patterns along the [001]* zone-axis^26^. (**b**) A 2-bit image (Ising system) converted from 8-bit data shown in Fig. 1(a). The figure contains 540 × 540 pixels. Three successive applications of 3 × 3 block-spin renormalisation were performed on Fig. 1(b) to yield Fig. 1(c)–(e).

PIN is an ideal system for the study of inherent randomness. In PIN, the arrangement of In and Nb can be controlled thermally, and the resulting structures are broadly classified into two categories: (1) a disordered PIN (D-PIN) in which In and Nb ions randomly occupy B sites in equal site numbers and (2) an ordered PIN (O-PIN) in which In and Nb ions are 1:1 ordered^23–25^. D-PIN exhibits relaxor behaviour with a freezing temperature at *T*_f_ ~ 240 K, whereas O-PIN exhibits a first-order antiferroelectric phase transition at *T*_N_ ~ 430 K. The fractal structure of D-PIN has been confirmed by electron-diffraction patterns, as shown in Fig. 1. Figure 1(a), obtained from ref.^26^, shows an inhomogeneous pattern. We converted this image to a 2-bit image (Ising system), as shown in Fig. 1(b), and we then applied three successive 3 × 3 block-spin renormalisations [Fig. 1(c)–(e)]. These pictures never exhibit a single, solid clour (black or white), indicating that the structure is self-similar. By comparing D-PIN with O-PIN, we can discuss the crossover from a normal phase transition to relaxor freezing behaviour and clarify the connection between random electric fields and the origin of relaxors with wide distributions of static and dynamic fluctuations.

## Results and Discussion

### Static properties of PIN: Diffuse X-ray scattering

**Figure 2:**
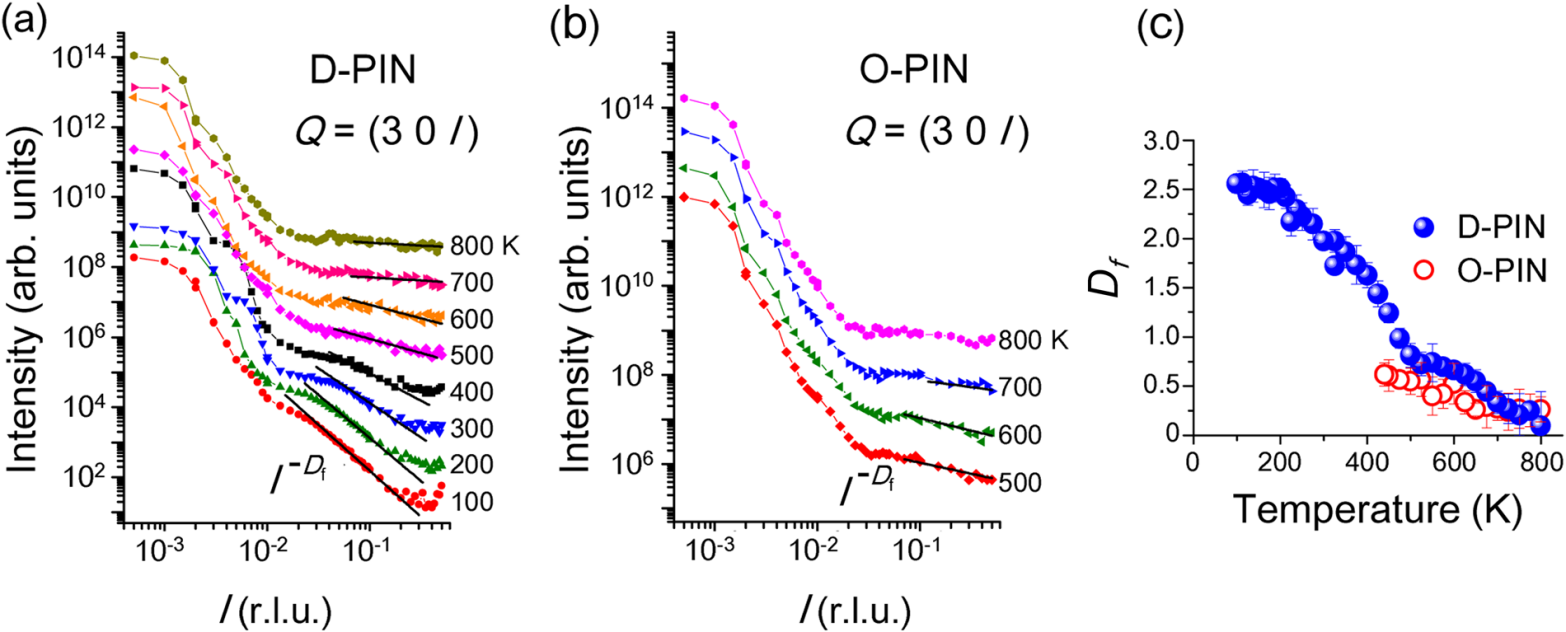
Diffuse scattering intensities along the transverse [001] direction near the 300 Bragg peak for D-PIN (**a**) and O-PIN (**b**). The straight lines show least-squares fits to the power law $I(l)=A{l}^{-{D}_{{\rm{f}}}}$, wherein *l* is in reciprocal lattice units (r.l.u.). The fractal dimension *D*_f_ are determined by the fits to the data shown in panels (a) and (b) and plotted as functions of temperature in panel (c). The fits were performed on ± *l*, and the averages are shown.

Figure 2(a) represents the diffuse X-ray scattering from D-PIN in the [00 *l*] direction around the 300 Bragg peak at various temperatures and on a double-logarithmic scale. At large *l* [between 0.033 and 0.19 reciprocal lattice units (r.l.u.)] and at 100 K [the red curve in Fig. 2(a)], the diffuse scattering is linear on a log–log plot, implying that it follows a power law, $I(l)\propto {l}^{-{D}_{{\rm{f}}}}$, with fractal dimension *D*_f_ = 2.55. This range of *l* corresponds to lengths ranging from 21 Å to 122 Å. These length scales and the value of *D*_f_ at 100 K are consistent with the low-temperature results for Pb(Mg_1/3_Nb_2/3_)O_3_^13^. The existence of self-similarity in D-PIN was also confirmed by our previous transmission electron microscope measurements and analysis, as shown in Fig. 1. As the temperature increases, the range of *l* becomes narrow and shifts to high values. Because the diffuse scattering in relaxors is mainly caused by the static component of the structure^27,28^, the power law results from the self-similar static PNRs. The change in *l*-range thus indicates that the distribution of static PNRs becomes narrow and that the average size of the PNR decreases upon heating. The fractal dimension *D*_f_ is plotted as a function of temperature in Fig. 2(c). It increases monotonically upon cooling, but the variation of *D*_f_ with temperature decreases below 220 K (~*T*_f_), slightly changing to the lowest measured temperature. The maximum value is approximately 2.5, which is close to the value *D*_f_ = 2.52 for a 3D percolation cluster. When the site-percolation threshold is set to 31.1%, 3D clusters due to percolation-by-invasion are calculated to have a fractal dimension ~2.52^29,30^.

It is widely accepted that relaxors are usually characterised by three temperatures. The highest is the Burns temperature *T*_B_^15^, above which the structure is essentially in the paraelectric state characteristic of normal ferroelectrics. Below *T*_B_, frustration between electric and spatial instabilities at the B site of the perovskite structure ABO_3_ induces dynamic PNRs (or nanometre-sized regions of time-dependent polarisation). The second temperature is the intermediate temperature *T* ^*^, below which some PNRs become sufficiently large to become static PNRs (or FNDs)^31,32^. The final temperature is the Curie temperature *T*_C_, at which the ferroelectric phase transition occurs [Alternatively, if the ferroelectric transition is absent, such as in D-PIN and Pb(Mg_1/3_Nb_2/3_)O_3_, the PNRs freeze at the temperature *T*_f_]^33^. Below *T*_C_, the static PNRs merge into larger microdomains^16^. The temperature dependence of *D*_f_ in D-PIN must reflect the growth process of inhomogeneous PNRs. Isolated PNRs at high temperatures (where *D*_f_ ~ 0) interact with each other, and their size increases with decreasing temperature, finally percolating in a 3D space below 220 K. The dramatic growth below 475 K must be attributed to the appearance of static PNRs (or FNDs), i.e. *T* ^*^ ~ 475 K. This behaviour of the diffuse scattering and the smooth change in *D*_f_ does not occur for homogeneous crystals. Relaxor ferroelectrics, however, are characterised by their inhomogeneous structures and growth processes.

Figure 2(b) shows the diffuse X-ray scattering patterns of O-PIN under the same experimental conditions as for Fig. 2(a). The diffuse scattering looks similar to that of D-PIN; however, the diffuse scattering is smeared below the antiferroelectric phase transition because *h*/4 0 *l*/4 superlattice reflections appear near the 300 Bragg reflection. At high temperatures, the temperature dependence of *D*_f_ for O-PIN shown in Fig. 2(c) is the same as for D-PIN; thus, we conclude that below *T*_B_, dynamic PNRs start to grow from a paraelectric phase in both D-PIN and O-PIN. The difference is that the growth of the fractal structure is interrupted by the antiferroelectric phase transition in O-PIN, whereas in D-PIN, the fractal structure grows until the inhomogeneous structure percolates in a 3D space.

### Dynamic properties of PIN: Light scattering

**Figure 3:**
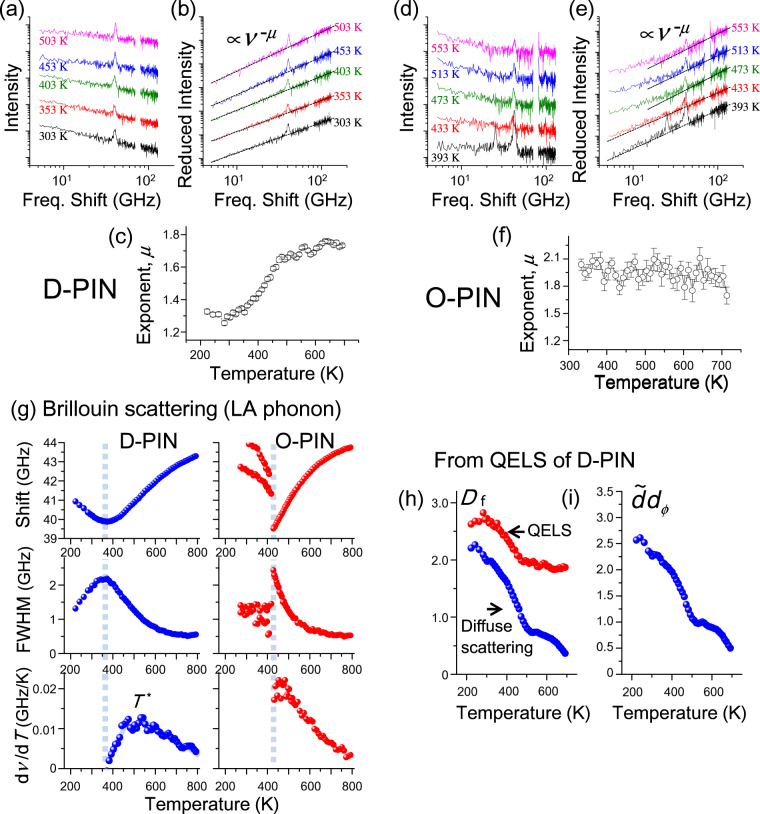
(**a**) Temperature variation of light-scattering spectra from D-PIN observed in the VH scattering geometry (see the Methods section). The QELS spectra follow the power law *I*(*ν*) ∝ *ν* ^−*α*^. (**b**) Reduced intensities *J*(*ν*) for D-PIN are calculated from *I*(*ν*) at selected temperatures. The dashed lines represent the least-square fits of *J*(*ν*) $\propto$ *ν* ^*μ*^. (**c**) The exponent* μ* for D-PIN as a function of temperature. (**d**) Temperature variation of light-scattering spectra from O-PIN observed in the VH scattering geometry. (**e)** Reduced intensity *J*(*ν*) for O-PIN calculated from *I*(*ν*). (**f**) The exponent *μ* for O-PIN as a function of temperature. (**g**) The frequency shift *ν*, FWHM *Γ*, and d*ν*/d*T* for Brillouin scattering from D-PIN and O-PIN. Panels (h) and (g) show parameters characterising the fractal structure in D-PIN as functions of temperature. The parameters *D*_f_ and $\tilde{d}{d}_{{\varphi }}$ were determined from diffuse X-ray scattering and light-scattering, respectively. The parameter *D*_f_ was also determined based only on light scattering in the same way as in ref.^14^, assuming $\tilde{d}$ = 1.32 and ${d}_{{\varphi }}$ = 1.

Light-scattering spectra from D-PIN and O-PIN are shown in Fig. 3(a) and (d) in a double-logarithmic plot. Scattering from longitudinal acoustic (LA) phonons appears as a Lorentzian-shaped peak near 40 GHz. The fitted parameters of the LA phonons in D-PIN and O-PIN, including the Brillouin shift *ν*, the full-width at half-maximum (FWHM) *Γ* and d*v*/d*T*, are shown in Fig. 3(g) as functions of temperature. The parameters *ν* and *Γ* show broad changes around the peak temperature of the temperature-dependent dielectric constant, i.e. *T*_m_ in D-PIN and *T*_N_ in O-PIN. The change is attributed to precursor phenomena before the freezing point of D-PIN and the antiferroelectric phase transition of O-PIN. When perovskite ferroelectrics do not contain any inhomogeneous structures, precursor phenomena are absent or appear only in a narrow temperature range with the LA phonons because a linear coupling between the order parameter and the longitudinal strain is prohibited in the paraelectric phases by symmetry restrictions^34,35^. Because the local structures in D-PIN and O-PIN are noncentrosymmetric, as shown by local-structure analysis^22^, the precursor phenomena can be attributed to piezoelectric coupling inside the noncentrosymmetric disordered regions, i.e. the PNRs. In other words, the polarisation fluctuations can couple linearly with the strain fluctuations in the inhomogeneous structures^10,36,37^. The ferroelectric instability in the antiferroelectric phase transition is not special but is also observed in PbZrO_3_^38^. The differences between the LA phonon in the paraelectric phases of D-PIN and O-PIN appear at the maximum point in d*v*/d*T* and at the minimum point in *dΓ*/*dT*, both at *T* ^*^, where (d*v*/d*T*)^−1^ and *dΓ*/*dT* can be interpreted as the growth rates of the polarisation fluctuations per degree K^10,37^. These differences between D-PIN and O-PIN indicate that the growth rate (temperature derivative) of the polarisation fluctuations in D-PIN increases during cooling but is suppressed below *T* ^*^ due to the appearance of the static PNRs, while the growth rate in O-PIN is not suppressed to *T*_N_. We postulate that the random electric fields in relaxor D-PIN generate the static PNRs in the paraelectric phase below *T* ^*^. It is worth noting that similar LA phonon behaviour was reported in disordered Pb(Sc_1/2_Nb_1/2_)O_3_ (D-PSN)^39^. Compared with D-PIN, D-PSN shows sharp changes at the phase transition. In future work, we will investigate the way in which different B-site ions tune the material’s properties.

The spectra of D-PIN in Fig. 3(a) show that QELS follows a power law, *I*(*ν*) ∝ *ν* ^*−α*^, wherein *ν* and *α* denote the frequency shift and exponent, respectively. Typically, the QELS follows a power law when the distribution of the relaxation time *τ* also follows a power law: *f*(*τ*) = *τ* ^−(*α* + 1)^ ^14,40^. Because the diffuse scattering shown in Fig. 2(a) is the result of the large size-distribution of self-similar PNRs, the relaxation time is distributed so that the PNR size is proportional to the relaxation time. The smooth change in *α* from 0.24 at 503 K to 0.73 at 303 K reflects the change in thermal excitation number of relaxation and the distribution of relaxation times. To remove the effect of the excited population from the light-scattering spectra, the reduced intensity *J*(*ν*) = *νI*(*ν*)/[*n*(*ν*) + 1] is usually used, wherein *n*(*ν*) is the Bose–Einstein factor. The reduced intensity *J*(*ν*) is proportional to *C*(*ν*) × *g*(*ν*), wherein *C*(*ν*) and *g*(*ν*) represent the light (Raman)-coupling function and the vibrational density of states, respectively. In other words, *J*(*ν*) contains information about the dynamics of the fractal structure— the fractons as well as the fluctuations of local strain^41,42^. The reduced-intensity spectra of D-PIN are shown in Fig. 3(b). The reduced intensity *J*(*ν*) also follows a power law [*J*(*ν*) ∝ *ν* ^*μ*^, where *μ* represents the exponent for a given reduced intensity spectrum].

Figure 3(c) shows how *μ* depends on temperature. As the temperature decreases from 700 K to 220 K, *μ* decreases from 1.8 to 1.3. The minimum in *μ* occurs at 280 K, which must be related to the fractal structure and its dynamics. The magnitude and temperature dependence of *μ* are consistent with the results of Koreeda *et al*.^14,40^.

The corresponding light-scattering spectra from O-PIN are shown in Fig. 3(d). The QELS can be reproduced by two power laws. The intensity is an order of magnitude smaller than that from D-PIN; thus, the low-frequency parts of the spectra may be due to a tail from the strong elastic scattering. Accordingly, we derived the exponent *μ* for O-PIN using just the high-frequency parts of the spectra, as shown in Fig. 3(e). Figure 3(f) shows the resulting value of *μ* as a function of temperature. The QELS are fairly independent of temperature and result in *μ* staying near 2. This indicates that the QELS originates from a phonon tail of a peak that occurs at a frequency greater than 1000 GHz. Reports indicate that in the scattering geometry, every perovskite-based relaxor ferroelectric has a phonon peak around 1000 GHz^21,43–45^. The peak shape is usually reproduced by a single Lorentzian or a damped harmonic oscillator; therefore the tail is proportional to *ν* ^−2^ at lower frequencies, as is observed in Fig. 3(e). Because *μ* is close to 2 for both D-PIN and O-PIN at high temperatures, the ferroelectric instability related to the QELS does not significantly contribute to the spectrum in either case at high temperatures. Only in D-PIN does the ferroelectric instability grow with decreasing temperature, which leads to an increase in QELS intensity and a decrease in *μ*. Inhomogeneous structures should exist in O-PIN, as expected from the LA phonons, but the value of *μ* for O-PIN indicates that the relaxation of these structures does not play an important role in this phase transition. This must be related to the fact that the antiferroelectric phase transition is induced by collective transverse-acoustic waves.

### Parameters characterising the fractals

Although *D*_f_, which represents the spatial self-similarity at a specific length scale, was determined from diffuse scattering experiments, two more parameters are needed to characterise a single fractal. The first is the fracton dimension $\tilde{d}$, which characterises a localised vibration at a fractal. The second is the localisation factor *d*_*ϕ*_, which determines the distance over which a single fracton can propagate in the strain direction: $u(r)\propto \exp [-\frac{1}{2}{(r/L)}^{{d}_{{\varphi }}}]$, wherein *L* denotes the localisation length. If *D*_f_ is determined as in refs^14^ and ^19^ with the two parameters fixed at $\tilde{d}=$ 1.32 and ${d}_{\varphi }=$ 1.0^14^, *D*_f_ increases gradually from 1.8 at 700 K to 2.6 at 223 K, as shown in Fig. 3(h). The quantity *D*_f_ behaves in a manner similar to that determined from diffuse scattering, except that the magnitude is different. We attribute this difference to the fact that the parameters $\tilde{d}$ and ${d}_{\varphi }$ were fixed in ref.^14^. The relation between *D*_f_, $\tilde{d}$ and ${d}_{\varphi }$ has been well-studied in previous investigations, which give the following relation^17,18,46^:

1 $$\mu =\tilde{d}\frac{2{d}_{\varphi }}{{D}_{{\rm{f}}}}-1,$$

which allows us to determine $\tilde{d}{d}_{\varphi }$ from light-scattering experiments and *D*_f_. (If ${d}_{\varphi }=\frac{{D}_{{\rm{f}}}}{\tilde{d}} \sim (2-\tilde{d})$ is valid as shown in ref.^18^, we can determine both $\tilde{d}$ and ${d}_{\varphi }$, independently. However, invalid ${d}_{\varphi }$ less than 1 was obtained using that equation in the present study. Similar problems were also pointed out in ref.^46^. Therefore, we only determined $\tilde{d}{d}_{\varphi }$). In this equation, *C*(*ν*) and *g*(*ν*) are proportional to ${L}^{{D}_{f}-2{d}_{\varphi }}$ and ${\nu }^{\tilde{d}-1}$, respectively. The product $\tilde{d}{d}_{\varphi }$ is evidently temperature dependent, as shown in Fig. 3(i). This indicates that a close connection exists between the distribution of spatial inhomogeneities and the distributions of characteristic times and/or localisations. This insight has not been pointed out in previous studies on relaxors. Because large static PNRs take long times to relax, we hypothesise that changes in $\tilde{d}$ dominate, rather than changes in ${d}_{\varphi }$. In other words, the static nature of the fractal structure in relaxors is linked to the dynamic characteristics of the PNRs.

### Impact of B-site randomness on inhomogeneity

**Figure 4:**
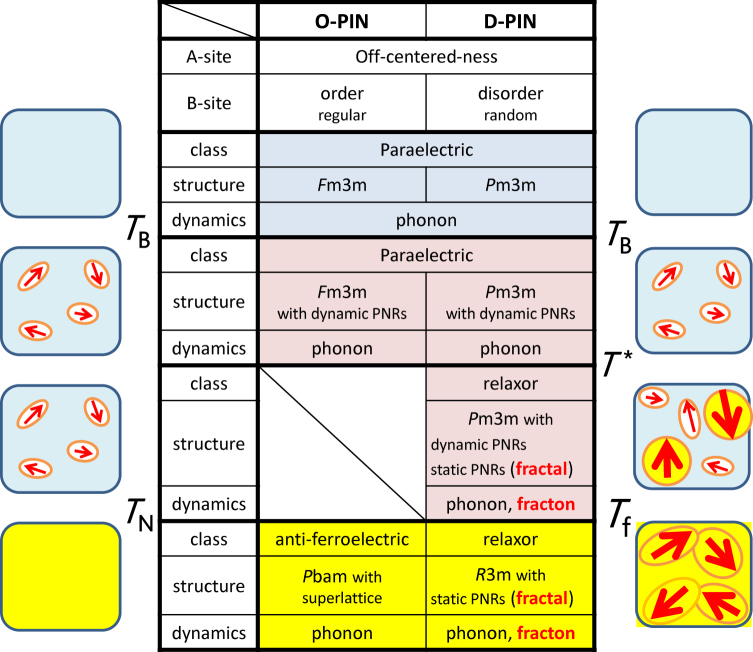
Comparison of static and dynamic properties of O-PIN and D-PIN. In D-PIN (on the right-hand side of the figure), the fractal structures of the PNRs grow as the temperature decreases below *T* ^*^ ~ 475 K, and nanosized ferroelectric domains are produced by percolation so that the PNRs become interconnected around *T*_f_ ~ 240 K. However, in O-PIN (left-hand side of the figure), the antiferroelectric phase is stabilised at *T*_N_ ~ 430 K, despite the ferroelectric instability in the paraelectric phase.

Based on the results from diffuse X-ray scattering and inelastic light scattering, we discuss below self-similar PNRs (or FNDs) with a wide distribution of characteristic lengths and times, as shown in Fig. 4. D-PIN and O-PIN differ from each other in the degree of disorder in the occupancy of their B sites. When In and Nb are disordered on the B sites, a fractal structure grows during cooling below *T* ^*^, and nanosized ferroelectric domains^47^ are produced by percolation around *T*_f_. In this case, the dielectric constant is enhanced by the localised vibrations (fractons). However, when In and Nb are ordered on the B sites, the zone-boundary transverse-acoustic (TA) phonon mode softens and induces the antiferroelectric phase at *T*_N_^48–50^. This is because short wavelength vibrations such as the zone-boundary TA phonons are easily affected by the occupation of the B sites. Given that the results for diffuse scattering, QELS and LA phonons are similar for D-PIN and O-PIN at high temperatures, we conclude that a ferroelectric instability always exists in this PIN system, but that ordered occupancy of the B-site stabilises the antiferroelectric phase, thereby suppressing long-range ferroelectric order and the growth of self-similar PNRs. One toy model, based on simple dipole-dipole interactions between off-centred Pb ions and Coulomb interactions from random B-sites^51^, distils the essence of the behaviour of D-PIN and O-PIN. It might be intriguing for future work to improve and apply this model tin an effort to produce the inhomogeneous structures with various temporal-spatial scales found in this study.

## Conclusion

In summary, we have determined the static and dynamic properties of B-site disordered Pb(In_1/2_Nb_1/2_)O_3_ and B-site ordered Pb(In_1/2_Nb_1/2_)O_3_ via diffuse X-ray scattering and inelastic light scattering. These properties depend on the degree of disorder of the B-site occupancy. When the B site is occupied randomly, the fractal structure grows as the temperature decreases below *T* ^*^ ~ 475 K, and nanosized ferroelectric domains are produced by the percolation of self-similar static polar nanoregions (PNRs). However, when the distribution of occupied In/Nb sites is regular, the antiferroelectric phase is stabilised, which suppresses long-range ferroelectric order and the growth of the self-similar PNRs. Understanding the inhomogeneous structures of ferroelectrics may enable enormous potential applications in high-performance capacitors and actuators and help to overcome the size effect of dielectric tips.

## Methods

To investigate the inhomogeneous structure in PIN crystals, we measured diffuse X-ray scattering around the 300 Bragg reflection along the transverse [001] direction over the wave-vector range from 0 to 0.5 r.l.u. at high resolution. Such a transverse scan does not detect strong Huang scattering, and this enables a simple discussion of the diffuse scattering from ferroelectric fluctuations in crystals^13^. The data were collected at temperatures from 100 K to 800 K with 12.398 keV X-rays at the BL22XU of SPring-8^52^. The energy was set just below the absorption-edge of Pb L_III_ to avoid the huge background due to X-ray fluorescence. The dynamics were probed by Rayleigh–Brillouin light scattering under a microscope and in the backscattering geometry^53^. To observe QELS from polarisation relaxation, the polarisation configuration was vertical-to-horizontal (VH); the vertical-to-vertical configuration detects not only QELS from polarisation relaxation but also that from thermal diffusion.

PIN single crystals were flux-grown from a PbO–In_2_O_3_–Nb_2_O_5_ system. The D-PIN crystals were prepared by quenching the as-grown crystals from 950 °C to room temperature, and the O-PIN crystals were prepared by annealing the D-PIN crystals at 923 °C for 20 h^24,25^. These crystals had dimensions of 2 × 2 × 1 mm^3^ and a pseudocubic (100) plane crystal habit. Because larger PIN crystals cannot be grown, and because indium has a large neutron-absorption coefficient, high-energy-X-ray and visible-light measurements are preferable to neutrons^48,49,53^. Before being measured, these crystals were etched with hydrofluoric acid (HF-etched) to remove the outer layers. Our previous study of *as-grown* PIN^51^ had found that the structure of the outer layers is different from that of the inner layers. We confirmed the removal of the outer layers with X-ray scattering using Mo *Kα* radiation at 17.5 keV. The temperature dependence of the complex dielectric constant of the PIN was also confirmed before the scattering measurements. Diffuse and frequency-dependent peaks were observed in D-PIN, but no frequency-dependent peaks were seen in O-PIN. The X-ray scattering and dielectric properties are shown in Supplementary Figure S1.

### Data Availability

The datasets generated during the current study are available from the corresponding author (S. T.) on reasonable request.

## Electronic supplementary material

Supplementary Information
