# Supplementary material for: Relation between Fractal Inhomogeneity and In/Nb-Arrangement in Pb(In1/2Nb1/2)O3
Source: Sci Rep. 2017 Dec 13;7:17508. doi: 10.1038/s41598-017-17349-3 (PMC5727483; doi:10.1038/s41598-017-17349-3)
Supplement: Supplementary file 1 — Supplementary Information [file 41598_2017_17349_MOESM1_ESM.doc]

**Relation between Fractal Inhomogeneity and In/Nb-Arrangement in Pb(In_1/2_Nb_1/2_)O_3_**

Shinya Tsukada^1,*^, Kenji Ohwada^2,†^, Hidehiro Ohwa^3^, Shigeo Mori^4^, Seiji Kojima^5^, Naohiko Yasuda^3^, Hikaru Terauchi^6^ & Yukikuni Akishige^7^

^1^ Faculty of Education, Shimane University, Matsue, Shimane 609-8504, Japan

^2^ National Institutes for Quantum and Radiological Science and Technology (in SPring-8), Sayo-cho, Sayo-gun, Hyogo 679-5148, Japan

^3^ Electrical and Electric Engineering Department, School of Engineering, Gifu University, Gifu 501-1193, Japan

^4^ Department of Materials Science, Osaka Prefecture University, Sakai, Osaka 599-8531, Japan

^5^ Pure and Applied Sciences, University of Tsukuba, Tsukuba, Ibaraki 305-8573, Japan

^6^ Advanced Research Center of Science, School of Science, Kwansei Gakuin University, Sanda, Hyogo 669-1337, Japan

^7^ Office of the Vice President for Research, Shimane University, Matsue 690-8504, Japan

**SUPPLEMENTARY INFORMATION**

The random and periodic arrangements of In and Nb in D-PIN and O-PIN were checked by single-crystal X-ray diffraction and dielectric-constant measurements. Figure S1 (a) shows the mesh scan results in the H0L zone for D-PIN and O-PIN (100) crystals at room temperature after HF-etching. The incident X-rays used were Mo *Kα* radiation at 17.5 keV. The absorption coefficient is close to that for (within at most a factor of 2) the 12.398 keV X-rays at BL22XU of SPring-8. Strong diffuse scattering around the Bragg points was clearly observed along the [101]-direction in D-PIN, which is a typical feature of lead-based perovskite relaxors, while *h* = 4, 0, *l* = 4 superlattice spots without any diffuse scattering were observed in the O-PIN. To determine the degree of order, we prepared 111-cut single crystals and the intensity ratio of the 1/2 1/2 1/2 and 111 Bragg diffractions was determined. The result for O-PIN shown in Fig. S1 (b) indicates that the degree of order *S*_2_^1^ is approximately 1; i.e. the In and Nb atoms are almost perfectly ordered. Conversely, in the case of D-PIN, we could not confirm the 1/2 1/2 1/2 reflection; i.e. the *S*_2_ value is approximately 0. The temperature dependences of the complex dielectric constants are also shown in Fig. S1 (c). Diffuse and frequency-dependent peaks were observed for D-PIN but not for O-PIN.

According to our previous study of *as-grown* PIN crystals^1^, the crystal structure of the outer layer is different from that of the inner layer; the outer layers show relaxor properties from diffuse scattering, while the inner layers show antiferroelectricity with superlattice reflections. Therefore, the results in Fig. S1 (a) obtained for D-PIN and O-PIN mean that such dual structures are removed by HF-etching after thermal annealing. In other words, the states of the prepared D-PIN and O-PIN samples at room temperature are a single-phase relaxor and a single-phase antiferroelectric, respectively. The removal of the outer layer was also investigated carefully and is discussed in Ref. 2.

^1^ Ohwada, K., Hirota, K., Terauchi, H., Ohwa, H. & Yasuda, N. Spatial distribution of the B-site inhomogeneity in an as-grown Pb(In_1/2_Nb_1/2_)O_3_ single crystal studied by a complementary use of X-ray and neutron scatterings. *J. Phys. Soc. Jpn.* **75**, 024606 (2006).

^2^ Ohwada, K., et al., Intrinsic ferroelectric instability in Pb(In_1/2_Nb_1/2_)O_3_ revealed by changing B-site randomness: Inelastic x-ray scattering study. *Phys. Rev. B* **77**, 094136 (2008).

Fig. S1: (a) Mesh scan results of D-PIN (left) and O-PIN (right). We observed strong, butterfly-shaped diffuse scattering for D-PIN and $h/4, 0, l/4$ superlattice spots for O-PIN. (b) Calculated and observed intensities of the 1/2 1/2 1/2 and 111 Bragg diffractions in O-PIN. (c) Temperature dependence of the complex dielectric constant of D-PIN and O-PIN. The probe frequency was from 75 kHz to 3 MHz.
